# Jet Splitting Enabled One-Step Fabrication of Hierarchically Structured PLA Membranes for High-Performance PM_0.3_ Filtration

**DOI:** 10.3390/nano15181452

**Published:** 2025-09-20

**Authors:** Yintao Zhao, Ying Chen, Xin Ning

**Affiliations:** 1 Industrial Research Institute of Nonwovens & Technical Textiles, College of Textiles & Clothing, Qingdao University, Qingdao 266071, China; zhytooo@163.com; 2 College of Textiles & Clothing, Dezhou University, Dezhou 253026, China; 3 Shandong Center for Engineered Nonwovens, Qingdao University, Qingdao 266071, China; 4 Qingdao Institute of Food and Drug Control, Qingdao 266071, China; cheny06301@163.com

**Keywords:** PLA air filter, hierarchical structure, jet-splitting electrospinning strategy, cationic antibacterial surfactant, high filtration performance, antibacterial performance, rapid heat dissipation

## Abstract

Particulate matter (PM) suspended in the air has posed significant potential threats to human health. However, current air filters designed to intercept PM are confronted with several challenges, including a complicated preparation process, monotonous protective performance, and uncomfortable wearability. Herein, a novel jet-splitting electrospinning strategy was demonstrated to simply fabricate a hierarchically structured PLA membrane with a high filtration performance, antibacterial performance, and rapid heat dissipation for effective and comfortable air filtering. Formulating a cationic antibacterial surfactant in the PLA solution to tailor the splitting of charged jets enables the simultaneous formation of nanofibers, submicron-fibers, and beads in the hierarchical filtration network by the single-jet electrospinning. Benefiting from the synergistic effect of multi-scale fibers and beads, the hierarchically structured filter exhibited an excellent filtration efficiency of 99.979% and high quality factor of 0.45 Pa^−1^ against PM_0.3_, with a remarkably low pressure drop of 18.7 Pa. Furthermore, the hierarchical structure endowed the filter with excellent stability in filtration performance, even under 20-cyclic and 480 min long-term tests, high-humidity tests with sodium chloride aerosol particles, and the 20-cycle PM_2.5_ smoke tests. Simultaneously, the filter also demonstrated remarkable antibacterial performance and an excellent heat dissipation property—all achieved due to its PLA formulation and the hierarchical structure.

## 1. Introduction

Particulate matter (PM) in the atmosphere has long been a significant global concern due to its direct impact on respiratory activity, which is fundamental to human life [1,2]. Particularly, PM represented by PM_0.3_ (aerodynamic diameter ≤ 0.3 μm) possesses characteristics of ultra-small volume, ultra-light weight, and highly penetrability, emerging as a significant perpetrator of cardiovascular and respiratory ailments, thereby posing substantial hazards to human health and even endangering life safety [3,4]. As a coping strategy, wearing an effective filter to prevent PM from being inhaled by the respiratory system is the simplest and most effective method [5,6]. Currently, the commercially available air filters commonly employ polypropylene-based melt-blown nonwovens as the filtration mediums [7]. However, due to the thick fiber diameter and large pore size, the filtration efficiency of melt-blown nonwovens heavily relies on electret charging [8]. These charges on melt-blown nonwovens easily experience dissipation when stored improperly or stored chronically, leading to a significant attenuation in filtration performance, thereby failing to effectively protect human health [9]. This effective charging strategy is also difficult to duplicate in a biobased filter system such as PLA, leading to less effective environmentally friendly technical options.

Electrospun nanofibrous filters provide a superior performance for air filters owing to their fine fiber diameters and tunable morphologies, which result in significantly smaller pore sizes compared to those of melt-blown nonwovens. These advantages enable them to achieve higher filtration efficiency, leading to new considerations in the field of air filtration [10,11,12]. However, these nanofibrous filters consist of small-diameter fibers that result in dense packing and subsequent elevated pressure drop, thereby causing breathing discomfort [13,14]. The crux of the issue lies in effectuating a delicate balance between filtration efficiency and pressure drop. To address this challenge, triboelectric and piezoelectric technologies, along with electret, have been introduced to fabricate nanofibrous filters that exhibit enhanced charge effect and PM capture capabilities, thereby achieving a high filtration efficiency and low pressure drop [15,16,17,18]. However, the dissipation of charge is also inevitable, especially in a high-humidity environment, thereby leading to a reduction in filtration efficiency.

A hierarchically structured nanofibrous filter appears to provide a satisfactory solution to achieve the balance between filtration efficiency and pressure drop, and large research efforts have been devoted to demonstrating the role played by hierarchical structures. For instance, an efficient and lightweight air filter with instant noodle-like curly nanofibrous structure has been prepared via the airflow and needleless electrospinning method. The as-prepared air filters showed a high PM_0.3_ removal efficiency of 99.1% along with a low pressure drop of 78 Pa [19]. A nanofibrous membrane with a wrinkled helical structure was fabricated using a combination of electrospinning and thermal treatment, employing polyvinyl alcohol/sodium alginate/hydroxyapatite as the raw materials. The resulting membrane demonstrated exceptional filtration efficiency of 99% against PM_0.3_ and low pressure drop of 95 Pa [20]. The sodium sulphobutylether-β-cyclodextrin/polyvinyl alcohol electrospun nanofiber membrane with typical curved-ribbon structure was developed by one-step electrospinning. The resultant membrane with hierarchical structure displayed a favorable filtration efficiency of 99.12% against PM_1.0_ and desirable pressure drop of 57.5 Pa [21]. Furthermore, favorable filtration performances were also achieved in hierarchical structures such as bead-on-string, nanofiber/nets, dual-scale fibrous networks, and other architectures [22,23,24,25,26]. The air filters prepared with the hierarchical structure exhibited exceptional filtration efficiency (>99%) and relatively low pressure drop (<200 Pa), owing to their inherent superiority in design. Nevertheless, there is still potential for further enhancement of the filtration performance while maintaining a satisfactory balance between filtration efficiency and pressure drop.

Moreover, as the public has gradually become apprehensive of the hazards of PM, wearing filters in outdoor environments has become a standard practice for them. Consequently, filtration performance is no longer the sole performance metric; the synergistic optimization of multifunctional health protection and wear comfort (particularly in high-temperature environments) is increasingly emerging as a core subject of research in air filters [24]. On one hand, PM exemplified by PM_0_._3_ with high specific surface areas can carry bacteria and viruses into the respiratory system, facilitating the rapid transmission of disease and widespread epidemics [27]. Notably, the representative COVID-19 outbreaks reportedly caused billions of infections and millions of fatalities worldwide, posing serious threats to public health, disrupting socioeconomic activities, and impeding global economic development [28,29]. However, even though most existing filters with painstaking design can capture the bacteria and viruses attached on the surface of PM, they fail to deactivate them. This allows bacteria and viruses to survive on filters, potentially causing secondary pollution [30]. On the other hand, as most filters achieved a high filtration performance at the cost of high thickness, their poor heat dissipation ability will become more evident in high-temperature environments. This limitation may consequently induce breath discomfort, lead to sudden health problems, and even result in irreversible serious consequences under thermal conditions [31].

Herein, we report a simple jet-splitting electrospinning strategy to construct, in one step, a hierarchical PLA-based nanofibrous filter featuring a high efficiency, low pressure drop, high antibacterial performance, and rapid heat dissipation. This method can overcome the limitations of existing filters, including complicated preparation processes, monotonous protective performance, and uncomfortable wearability. By introducing a cationic antibacterial surfactant into the PLA solution at a low concentration, splitting of the charged jets was induced due to the variations in solution properties, particularly the conductivity, enabling the simultaneous formation of nanofibers, submicron-fibers, and bead structures in the hierarchical nanofibrous filter. Under the influence of the providential synergistic effect brought on by the multi-scale fibers and beads, the hierarchical fibrous filter exhibited an outstanding filtration efficiency and low pressure drop. The filtration performance of the filter maintained excellent stability, even during cyclic tests, long-time tests, tests in incremental airflow velocities, continuous tests at high-humidity conditions, and real tests against actual PM_2.5_ smoke. Moreover, the hierarchical nanofibrous filter showed superior antibacterial performance owing to the effective antibacterial contents in the fibers. The lightweight and thin filter also possessed better heat dissipation performance compared to commercial filters. Overall, this work demonstrated a new way of developing environmentally friendly filtration materials with high-protective performance and comfortable wearability.

## 2. Materials and Methods

### 2.1. Materials

PLA (Ingeo^TM^ 4032D, *M*_w_ = 15.6 × 10^4^ g/mol) was purchased from NatureWorks LLC (Minnetonka, MN, United States). 2,2,2-Trifluoroethanol (TFE) and Dodecyltrimethyl ammonium chloride (DTAC) were provided by Shanghai Macklin Biochemical Technology Co., Ltd. (Shanghai, China). PLA spunbond nonwoven fabric with base weight of 20 g·m^−2^, filtration efficiency below 5%, and pressure drop of 1–2 Pa for sample collection and testing was provided by Smmartin Import and Export Trading Co., Ltd. (Quanzhou, China).

### 2.2. Preparation of Nanofibrous Membranes

The homogeneous solutions were prepared by dissolving PLA, or PLA and DTAC with varied ratios, into TFE under continuous stirring at ambient temperature for 6 h; the corresponding details were recorded in Table S1. The as-prepared solutions were then sucked into the 5 mL syringes assembled with 21-gauge needles for high-voltage electrospinning, with the feeding rate of 1 mL/h, the working distance of 18 cm, the fixed voltage of 25 kV, the temperature of 28 ± 1 °C, and the relative humidity of 55 ± 5%, to prepare PLAx and PLAx/DTACy, where “x” represents the weight percentage (wt%) of PLA (x= 2, 4, 6, 8) to solvent, and “y” indicates the weight percentage (wt%) of DTAC to PLA (y = 1, 3, 5, 7). The basis weights of samples were regulated by adjusting the spinning time.

### 2.3. Characterization

The variation trend of conductivity in solutions was measured using a DDSJ-308F conductivity meter (Shanghai Instrument & Electronics Science Instrument Co., Ltd., Shanghai, China). The viscosity of solutions was evaluated by a RVTV-2H digital rotary viscometer (Shanghai Pingxuan Scientific Instrument Co., Ltd., Shanghai, China). A KRUSS K100 (KRUSS Scientific Instruments Co., Ltd., Hamburg, Germany) was employed to determine the surface tension of solutions. The microstructures of samples and their cross section were characterized using a Tescan Mira LMS scanning electron microscopy (Tescan Ldt., Brno, Czech Republic). Nanomeasure (version 1.2) and ImageJ software (version win64) were employed to measure the fiber diameters, cross-section thicknesses and pore sizes of samples referring to the method reported in our previous work [32]. A Nicolet iS10 FTIR spectrometer (Thermo Fisher Scientific, Waltham, USA) equipped with an attenuated total reflectance (ATR) accessory was employed to test the fourier transform infrared (FTIR) spectra of samples in the range of 4000–500 cm^−1^ with 32 scans at a resolution of 4 cm^−1^.

The basis weights of samples were measured by SQP QUINTIX35-1CN balance (Sartorius Corp., Goettingen, Germany). The thermal images of the as-prepared sample and the other commercial filters were shoot by a TiS20+ Thermal Imager (Fluke, Everett, WA, USA).

### 2.4. Filtration Tests

The filtration performances of the samples were evaluated using an AFC-131 filter tester (Topas GmbH, Germany) with an effective filtration area of 176 cm^2^. A charge-neutralized sodium chloride (NaCl) aqueous solution with the concentration of 3 wt% was used as particle source, and the solution was atomized into a large amount of PM with suitable particle size distribution by a particle generator. Then, the PM entered into the filter duct under controlled airflow. The PM concentrations upstream and downstream of the filter material were measured using integrated particle counters. The filtration performances of samples were measured at a constant airflow rate of 32 L·min^−1^. The pressure drop (Δ*P*, Pa) was determined by measuring the pressure differences between the upstream and downstream airflows through the differential pressure sensor, and the determination of filtration efficiency (*E*, %) abided by the following Equation (1) [8]:

(1) $$E=1-\frac{N_{down}}{N_{up}}$$

where *N*_down_ and *N*_up_, respectively, represent the particle number in the downstream and upstream, as gauged by the filter tester. Quality factor (*QF*) as a comprehensive indicator for evaluating the comprehensive filtration performance of air filters can be calculated by the following Equation (2) [33]:

(2) $$QF=\frac{-ln(1-E)}{\Delta P}$$

To verify the practical application potential, the filtration efficiencies of samples against PM_2.5_ generated by the burning sandalwood were measured for 20 cycles in a self-made circulating filtration device with a PM_2.5_ detector. The real-time PM_2.5_ contents displayed on the PM_2.5_ detector were recorded per 10 s until the concentration dropped below 10 μg/m^3^, while the initial concentrations were set as over 900 μg/m^3^.

### 2.5. Antibacterial Tests

The shake flask method was employed to quantitatively assess the antibacterial activity of the as-prepared samples according to GB/T-20944.3-2008 [34]. Initially, 0.75 g of the sample was added to the bacterial suspension and incubated in a shaker at a constant temperature of 37 °C for 24 h. Next, 200 μL of appropriately diluted bacterial suspensions were uniformly smeared on the surface of agar plates. The Petri dishes were then transferred to a constant temperature incubator at 37 °C and incubated for an additional 24 h. Finally, the antibacterial efficiency was determined by calculating the ratio of colony-forming units (CFUs) in the experimental group relative to those in the control group.

## 3. Results and Discussion

### 3.1. Design and Preparation of Hierarchical Fibrous Filter

**Figure 1:**
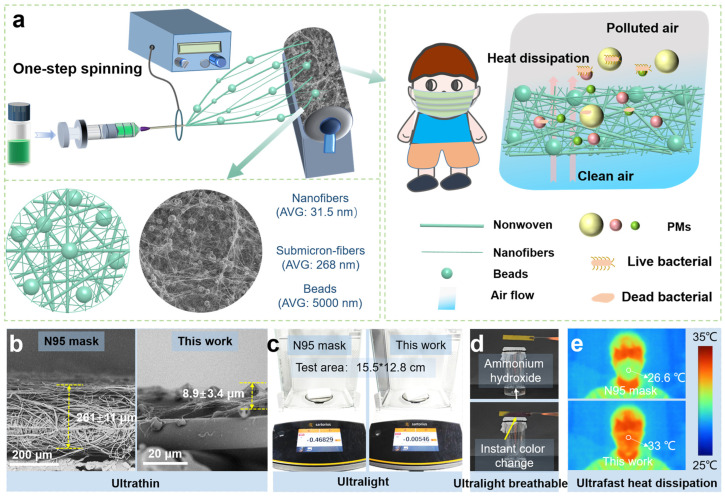
(**a**) Schematic diagram of one-step electrospinning process, and structural and filtration characteristics of the hierarchical nanofibrous filter. (**b**) SEM images comparing the cross-section thickness of filtering layer in N95 mask and hierarchical nanofibrous filter. (**c**) Weight of filter layer in N95 mask and hierarchical nanofibrous filter with the test area of 15.5 cm ×12.8 cm. (**d**) The breathability of hierarchical nanofibrous filter. (**e**) Heat dissipation of N95 mask and hierarchical nanofibrous filter.

To achieve the facile and rapid fabrication of an air filter with multidimensional and comfort protective properties, we designed the filter based on the subsequent criteria: (i) The fibrous filter should feature a hierarchical structure to achieve high filtration efficiency against the high-permeable PM_0.3_, while maintaining a low pressure drop. (ii) The fibrous filter should be prepared simply and conveniently in one step to ensure cost-effectiveness and production efficiency, while exhibiting lightweight and ultra-thin structures to ensure comfortable wearability. (iii) The fibrous filter should demonstrate effective antibacterial ability to avoid the proliferation of bacteria and viruses, thereby achieving multi-protection. To satisfy the above criteria, the PLA solutions of varied concentrations below the entanglement concentration (*Ce*) of 10.2 wt% calculated in previous work [32] were spun to select the suitable concentration for preparing a hierarchically bead-on-string structure. The structure, as a three-dimensional space structure, could provide more airflow passage in the filter, thereby assisting in the achievement of a high filtration efficiency and low pressure drop, and additionally ensuring heat transfer and comfortable wearability [5]. Additionally, dodecyltrimethylammonium chloride (DTAC) as a cationic antibacterial surfactant was imported in electrospinning solution to tailor the ejection of the charged liquids [24,25]. It can help improve the charge density by ionizing ions, thereby inducing the further splitting of jets [25]. Then, the jets undergoing splitting were drawn into a favorable scale of fibers (nanofibers with an average diameter of 31.5 nm; submicron-fibers with an average diameter of 268 nm) and beads (with an average diameter of 5000 nm) by one step. Therefore, the preparation process of the filter was commendably simplified together with the spinning time, which was also economized (Figure 1a). The multi-scale fibers, beads, and favorable pore size ensured the superior filtration efficiency of the hierarchical structured filter, providing clean air for human respiration. Meanwhile, the heat generated by respiratory activities was more likely to pass through the filter to ensure comfortable wearability. Additionally, through contact or release effects, DTAC in the filter could interact robustly with the lipid bilayer of the microbial membrane, destroying the bacterial membrane and ultimately leading to bacterial death [35,36], thereby achieving multi-protection for human beings.

Owing to the well-designed complex hierarchical structure, the filter with high filtration performance possessed an ultra-thin thickness of only 8.9 μm, while the thickness of the filter layer in commercial medical mask was as high as 261 μm (Figure 1b). The weight of our filter was merely 0.00546 g (testing area was 15.5 cm × 12.8 cm), while the corresponding weight for the filter layer in commercial medical masks amounted to a staggering 0.46829 g—approximately 86 times heavier than our filter (Figure 1c). An instantaneous color change in the PH paper when close to ammonium hydroxide covered by the filter demonstrated the exceptional breathability of our filter, attributed to its remarkably thin structure and three-dimensional support framework reinforced by beads that facilitated unhindered gas permeation through the filter (Figure 1d). The complex structure of the filter also facilitated efficient dissipation of the heat generated during respiration to the outside environment, resulting in a filter surface temperature as high as 33 °C during exhalation, while the N95 mask surface temperature was only 26.6 °C (Figure 1e).

### 3.2. Morphology and Structure of Hierarchical Nanofibrous Filters

To prepare the robust, protective, and comfortable nanofibrous filters with favorable hierarchical morphology, the scheduled concentrations of PLA solutions were initially set as 2, 4, 6, and 8 wt% to screen the suitable concentration for obtaining the bead-on-string structure. At the extremely low concentration of 2 wt%, a significant number of individual beads with varying sizes were observed (Figure S1a). However, no fiber formation was evident, which could be attributed to the low viscosity of the solution at this concentration, which failed to balance the electrostatic repulsion and counteract the shrinkage caused by surface tension [5,37]. The electrospinning process was substituted with electrospray, leading to the production of a substantial quantity of micron-sized beads. Upon increasing the concentration to 4 wt% (Figure S1b), a higher degree of chain entanglement in the solution weakened the electrostatic repulsion and partially mitigated the shrinkage of surface tension, resulting in a limited occurrence of ultra-fine fibers between beads in PLA4 [38,39]. With a further increase in concentration to 6 wt%, an increased number of fibers were observed in PLA6, accompanied by a significant decrease in the number of beads, which exhibited spindle shapes to some extent (Figure S1c). The 8 wt% concentration approached the *Ce* threshold, so the PLA chain entanglement was enhanced, thereby resulting in the further reduction in beads (Figure S1d). Considering that the number of beads decreased with the increase in PLA solution concentration, no fiber formed at the extremely low concentration of 2 wt%, and DTAC contributes to conductivity augmentation in higher concentrations of electrospinning solutions, we selected a concentration of 4 wt% PLA solution with varying contents of DTAC for subsequent experiments to achieve the coexistence of beads and further formed fibers.

**Figure 2:**
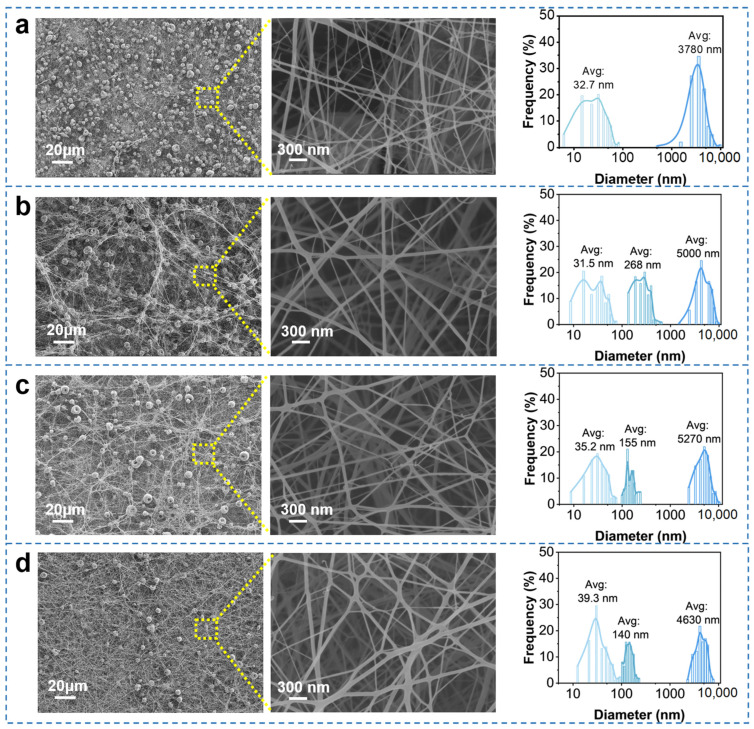
Microscopic structures, partial enlarged detail, and diameter distribution of (**a**) PLA4/DTAC1, (**b**) PLA4/DTAC3, (**c**) PLA4/DTAC5, and (**d**) PLA4/DTAC7.

While the surface morphology of hierarchical nanofibrous filters made from 4 wt% PLA solution exhibited a substantial amount of bead structure and a minor presence of fibers (Figure S1b), the involvement of 1 wt% DTAC in solution resulted in an increased fiber structure (Figure 2a), although the bead structure still dominated in the membrane. The average diameter of the beads in PLA4/DTAC1 was 3780 nm, while the average diameter of the merely formed nanofibers was 32.7 nm, showing a bimodal structure, and a certain proportion of the nanofibers even had diameters lower than 10 nm. As the content of DTAC increased to 3 wt%, the bead structure no longer dominated in PLA4/DTAC3 but coexisted equally with the fiber structure (Figure 2b). The average diameter of the beads increased to 5000 nm. Simultaneously, the number of fibers further increased, and submicron-fibers with an average diameter of 268 nm and nanofibers with an average diameter of 31.5 nm emerged. Notably, extremely fine fibers with diameters less than 10 nm were also present. Benefiting from the combination of multi-scale fibers and an appropriate amount of beads, the PLA4/DTAC3 exhibited a fluffy structure, which was beneficial for obtaining a smaller pressure drop. The fiber structure became dominant with the further increased DTAC content of 5 wt% (Figure 2c), as the beads no longer appeared to be largely distributed in PLA4/DTAC5. The increase in average diameter of beads to 5270 nm can be attributed to the reduction in the number of small-sized beads, and the solution was released faster from the needle under the influence of DTAC. Moreover, the nanofibers, with an average diameter of 35.2 nm, and the submicron-fibers, with an average diameter of 155 nm, also were closely related to the faster ejection speed and the influence of DTAC on jet stretching. As the DTAC in PLA solution concentration increased to 7 wt% (Figure 2d), the dominance of the fiber structure became even more prominent, accompanied by a sharp decrease in the bead structure. The significant reduction in volume of the bead structure (average diameter reduced to 4630 nm) could be ascribed to the fact that the ions ionized by DTAC increased the charge density, and the enhanced charge self-repulsion in PLA solution facilitated the charged jets stretching into fibers [40]. Additionally, the transformation of more jets into fibers influenced the distribution of nanofibers, while the average diameter of the submicron-fibers decreased to 140 nm, primarily due to the higher charge self-repulsion and stretch force. The chemical structure of PLA4, DTAC, PLA4/DTAC1, PLA4/DTAC3, PLA4/DTAC5, and PLA4/DTAC7 was analyzed by FTIR measurements to verify the effective combination of DTAC and PLA (Figure S2). The characteristic peaks of PLA4 appeared at 2996 cm^−1^ and 2944 cm^−1^, corresponding to the asymmetric and symmetric stretching of C-H, respectively, while the peaks at 1750 cm^−1^, 1450 cm^−1^, and 1351 cm^−1^ represented the stretching of C=O, asymmetric bending of CH_3_, and symmetric bending of CH_3_, respectively. The characteristic absorption peaks at 1180 cm^−1^, 1128 cm^−1^, and 1086 cm^−1^ were attributed to the asymmetric stretching of C-O-C, stretching of O-C=O, and the symmetric stretching of C-O-C. The bending of OH, the stretching of C-C, and the bending of C=O were observed at 1041 cm^−1^, 869 cm^−1^, and 756 cm^−1^, respectively. The characteristic peaks of PLA4 were consistent with those of pure PLA reported in the literature [32,39], and all of them were also present in the DTAC-doped PLA membrane, indicating that the addition of DTAC did not alter the functional group distribution of PLA. The DTAC-doped PLA membranes exhibited characteristic peaks inherited from pure DTAC, where the peak at 2920 cm^−1^ corresponded to the stretching vibration of –CH_3_, and the peak at 2850 cm^−1^ was attributed to the stretching vibration of –CH_2_ [41]. This confirmed that DTAC had been effectively incorporated into the PLA membrane.

### 3.3. Solution Properties and Structural Formation Mechanism

**Figure 3:**
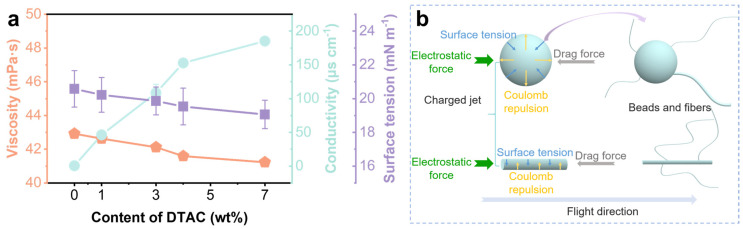
(**a**) Viscosity, conductivity, and surface tension of 4 wt% PLA solutions with different contents of DTAC. (**b**) Schematic diagram of the forces acting on charged jets and droplets during flight and their deformation process.

To clearly study the influence of DTAC content on the formation mechanisms of fibers and beads in the membrane, the properties of 4 wt% PLA solution with varying DTAC contents were measured and analyzed (Table S2, Figure 3a). As the content of DTAC increased from 0 wt% to 7 wt%, there was a slight reduction in viscosity from 42.92 mPa·s to 41.23 mPa·s, a mild decrease in surface tension from 20.58 mN·m^−1^ to 19.06 mN·m^−1^, and the conductivities showed a remarkable increase from 0.56 μs·cm^−1^ to 185.25 μs·cm^−1^. These phenomena can be attributed to the fact that DTAC, as a small molecule substance, can act as a plasticizer between the PLA molecular chains, promoting the sliding of the molecular chains and preventing their physical cross-linking. Then, the intermolecular forces between the PLA chains in the solution were weakened, thereby decreasing the solution viscosity and surface tension. Moreover, as a quaternary ammonium salt, DTAC can ionize into positively charged dodecyl trimethylammonium cations and negatively charged chloride ions when dissolved in the solvent, thereby increasing the charge density and enhancing the electrical conductivity in the solution. When the DTAC concentration was 1 wt%, the solution’s conductivity, the charge density, and the Coulomb force also increased sharply under the influence brought on by the ionization of DTAC. Meanwhile, the forces exerted by surface tension and viscosity weakened. Therefore, it became easier for the charged jets to break free from the constraint of surface tension and split into smaller jets or droplets under the influence of Coulomb repulsion (Figure 3b). Then, the fine fibers or beads were formed through the rapid stretching effect of the electric field force [42]. The conductivity further increased when the concentration of DTAC increased to 3 wt%, together with the further decrease in viscosity and surface tension. This enhanced the phenomenon of jet splitting induced by Coulomb repulsion. The split jets then underwent stretching by the electric field to form fibers with varying thickness. At the same time, droplets were also split into smaller droplets and fine jets under the influence of Coulomb repulsion; this was probably the main reason why submicron-fibers begin to appear in the membrane. Subsequently, droplets and jets continued to undergo stretching under the stretching force of the electric field to form fibers or beads. Therefore, under the influence of the enhanced electric field stretching force and the enhanced jet split effect, the number of fibers gradually increased, while the number of beads continuously decreased. Moreover, the enhancement of Coulomb repulsion also enabled the jets to separate from the Taylor cone more quickly, which was conducive to the formation of larger volume bead structures [43,44]. The concentration of DTAC further increasing to 5 wt% and 7 wt% indicated that more DTAC molecules had undergone ionization in the solution, thereby causing an increase in the charge density of the solution [45,46] and leading to a further enhancement of the dominant role of Coulomb repulsion in the charged jets. Those factors, together with the further weakened surface tension and viscosity, resulted in an increasing number of fiber structures and a gradual decrease or even disappearance of bead structures [47,48]. As for the thicker nanofibers and thinner submicron-fibers in the membrane, they were both caused by the combined effects of the enhanced stretching force from the electric field, the stronger jet splitting, and other factors.

Overall, the addition of an appropriate amount of DTAC can induce jet splitting, enabling the one-step formation of multiple structures. This approach not only simplified the fabrication process of filters but also endowed the filters with a hierarchical structure, thereby enhancing their air filtration performance. Therefore, the as-prepared PLA4/DTAC3, which was composed of multi-scale fibers and three-dimensional beads with a moderate distribution proportion, can effectively prevent the excessive close packing of various structures, which is more suitable for achieving high filtration performance from a structural perspective. Consequently, PLA4/DTAC3 worked well with our design for the filter, and can be utilized for subsequent research and testing.

### 3.4. Spinning Time Effect on Filters and the Filtration Mechanisms

**Figure 4:**
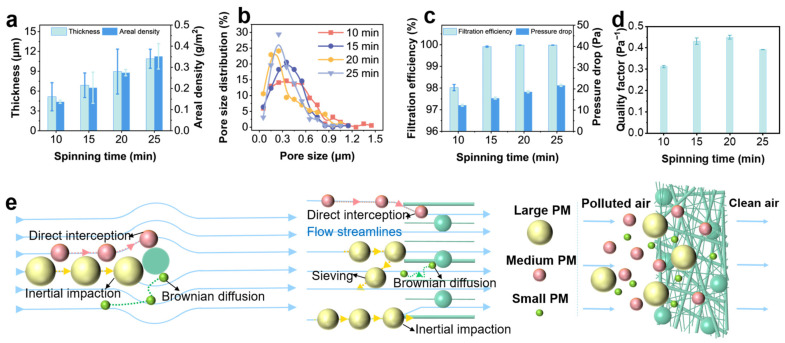
(**a**) The thickness and areal density, (**b**) pore size distribution, (**c**) filtration efficiency and pressure drop, and (**d**) *QF* value at different spinning times. (**e**) Scheme illustrating the filtration mechanisms existing in the hierarchically structured PLA4/DTAC3.

The spinning time is the key factor determining the thickness, basic weight, and filtration performance of the filter. Therefore, the spinning time was set within the range of 10 to 25 min, and the parameter variations resulting from the accumulation of spinning time were collected (Table S3). The thickness of PLA4/DTAC3 demonstrated an increase from 5.15 μm to 10.9 μm as the spinning time increased (Figure 4a, Table S3). Based on the analysis of structural characteristics in the filter, it can be inferred that the substantial increase was primarily attributed to the presence of beads, whose diameters were considerably larger than those of the fibers, and it possessed a three-dimensional structure with excellent support properties. The basic weight also exhibited a progressive increase from 0.137 g·m^−2^ to 0.352 g·m^−2^, maintaining a linear correlation with time, which can be attributed to the continuous deposition of fibers and beads on the collector device. Due to the mutual complementarity among the multi-scale fibers and beads, the average pore size of the PLA4/DTAC3 was as small as 0.45 μm, even when the spinning time was only 10 min (Figure 4b). As the spinning time increased, the pore size decreased and eventually stabilized at 0.34 μm when the spinning time reached at 20 min. However, further extension at a spinning time of 25 min showed no statistically significant variation in pore size. The filtration efficiency of filters increased from 97.88% to 99.979% toward PM_0.3_, while the pressure drop increased from 12.3 Pa to 21.7 Pa when the spinning time accumulated. When the spinning time was 20 min, the filtration efficiency reached the same level as that at 25 min (Figure 4c), while the pressure drop at this time was smaller than that at 25 min, which was attributed to the fact that the areal density at 25 min exceeded the optimal value. The *QF* values initially increased with the extension of spinning time, reaching the optimal value at 20 min of 0.444 Pa^−1^. Subsequently, the *QF* values declined as spinning time continued to increase (Figure 4d). This trend was closely correlated with the changes in filtration efficiency, which ceased to improve beyond the optimal spinning time, while the pressure drop continuously increased. Therefore, the PLA4/DTAC3 spun for 20 min was compared with the literature to evaluate the superiority in filtration performance (Figure S2), and was utilized for subsequent analyses. Comparatively, the PLA4/DTAC3 exhibited superior filtration efficiency to the other filters, while maintaining significantly lower pressure drop, indicating its exceptional practical application potential.

Based on the classical filtration theory, the filtration efficiency achieved by fiber materials can be attributed to five mechanisms, including Brownian diffusion, direct interception, inertial impaction, gravitational deposition, and electrostatic deposition [49,50]. These mechanisms usually exert synergistic effects in actual filtration processes, affecting the filtration performance of filters. At the same time, these mechanisms are influenced by the microstructure of filters and achieve differential contributions. Analyzing the filtration mechanism through the pore size perspectives, the complex and hierarchical assembly of multi-scale nanofibers and beads in PLA4/DTAC3, achieving an average pore size of 0.34 μm smaller than that of most large PM, ensured optimal utilization of direct interception, Brownian diffusion, and inertial impact mechanisms. Revealing the filtration mechanisms from hierarchical structural perspectives, the nanofibers in PLA4/DTAC3, with fiber diameters below 100 nm, provided the PM with a substantial specific surface area to achieve attachment, thereby significantly increasing the probability of PM capture through the mechanisms of inertial impaction or Brownian diffusion [51]. These ultra-fine fibers were capable of interweaving with each other, cutting through the original pathways of PM and greatly enhancing the probabilities of interception. The submicron-fibers of 268 nm, with diameters comparable to PM_0.3_, could also offer abundant surface area for PM. They collaborate with the nanofibers to create multiple obstacles for PM passage, improving the capture probabilities via mechanisms such as direct interception, inertial impaction, and Brownian diffusion [52] (Figure 4e). The three-dimensional bead structure, through its spatial support within the PLA4/DTAC3, spatialized airflow pathways, improving the breathability of the fiber membrane while also providing attachment points for PM retention [53]. On the whole, PLA4/DTAC3 exhibited a high filtration efficiency, owing to the synergistic effects of multiple mechanisms and their well-designed hierarchical structures.

### 3.5. Filtration Stability of PLA4/DTAC3

**Figure 5:**
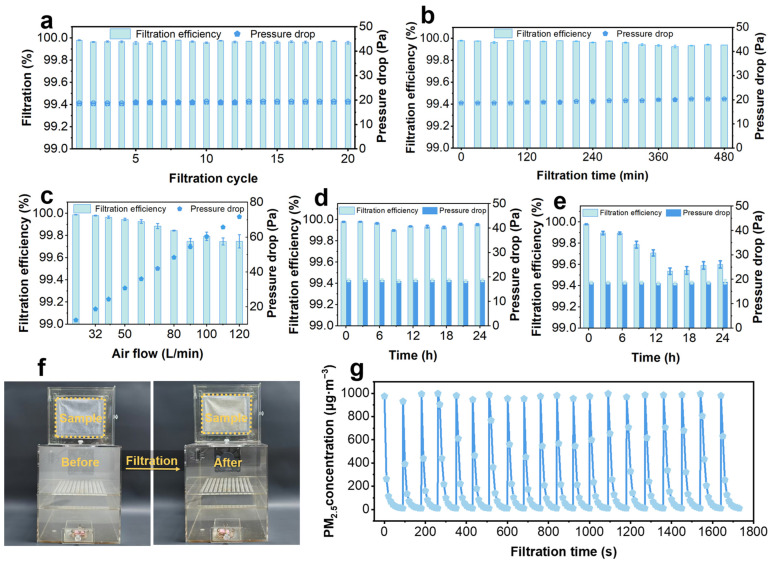
Filtration efficiency and pressure drop of PLA4/DTAC3 during (**a**) cyclic test for 20 times, (**b**) continuous long-time test for 480 min, (**c**) tests under different airflow velocities, (**d**) continuous test at 60% RH, and (**e**) continuous test at 90% RH. (**f**) Images showing PM_2.5_ in the box before and after being purified by PLA4/DTAC3. (**g**) Consecutive 20-cycle filtration tests of PLA4/DTAC3 against actual PM_2.5_ smoke.

The stability of filtration performance in filters is essential, as it determines the reliability of the filters in practical applications. To systematically evaluate the stability and durability of filtration performance, PLA4/DTAC3 were tested in various conditions. During a 20-cycle filtration test, the filtration efficiency and pressure drop of PLA4/DTAC3 were both excellently kept around their initial values with negligible variations (Figure 5a and Table S4). Meanwhile, a similar phenomenon was also identified during the continuous long-time filtration tests for 480 min (Figure 5b), together demonstrating the outstandingly stable filtration performances of PLA4/DTAC3. Notably, after continuously testing for 480 min, the pressure drop increment of PLA4/DTAC3 was only 1.6 Pa, while the filtration efficiency of PLA4/DTAC3 had reduced to 99.939% toward PM_0.3_ (Table S5), probably attributed to the fact that the continuous air flow has a slight effect on the structure of ultra-thin PLA4/DTAC3, or the wet sodium chloride aerosol weakened the electrostatic effect of the PLA4/DTAC3.

A nearly linear increase trend in the pressure drop of PLA4/DTAC3 with the growing airflow rates was observed (Figure 5c), keeping consistent with previous reports [54,55], while their filtration efficiencies were hardly influenced by the significantly increased airflow, indicating a favorable performance stability of PLA4/DTAC3. Even when the airflow rates increased by 500% from 20 L/min to 120 L/min, the filtration efficiency of PLA4/DTAC3 merely decreased by 0.24% from 99.987% to 99.747% (Table S6), and the slight efficiency loss probably resulted from the reduced retention time of PM at a high airflow rate, diminishing the filtration efficiency through Brownian diffusion [56].

The filtration performance of filters may significantly descend in high-humidity environments due to the influence of airborne water molecules. Therefore, the filters should possess a stable filtration performance under high-humidity conditions to maintain high-protection. Therefore, PLA4/DTAC3 was placed in a sealed high-humidity chamber and tested every 3 h for a continuous 24 h to evaluate its stability of filtration performance. After exposure to a 60% RH environment for a consecutive 24 h, the filtration efficiency of PLA4/DTAC3 remained at a high level of 99.953%, with no significant change in pressure drop (Figure 5d, Table S7). The stability can be attributed to the fact that moisture content in the air remained gaseous without condensing into liquid droplets. Consequently, the structure of the PLA4/DTAC3 remained unaffected, ensuring unimpaired filtration performance. However, when the PLA4/DTAC3 was placed in a 90% RH environment (Figure 5e), the filtration efficiency of PLA4/DTAC3 exhibited small fluctuations, characterized by an initial decrease followed by an increase. After 24 h, the filtration efficiency was measured as 99.6%, still maintaining a high level, while the pressure drop increased by 0.3 Pa (Table S8). These phenomena can be attributed to the fact that the lightweight and thin structure of the PLA4/DTAC3 made it easier for its three-dimensional structure to be influenced by the dense water vapor, resulting in a minor collapse in the filter structure. This contributed to the slight decrease in filtration efficiency and the negligible increase in pressure drop. The subsequent recovery in filtration efficiency after 15 h can be attributed to the improved wettability of the fiber surfaces, increasing the adhesion toward PM during filtration, thereby improving filtration efficiency. In summary, although PLA4/DTAC3 exhibited a slight performance degradation under extremely high humidity, it still showed exceptional protective performance.

The stability of filtration performance in practical applications is also an important parameter, as it can better reflect the protective performance of the filter in actual employment. To mimic practical applications, the filtration performances of PLA4/DTAC3 against actual PM_2.5_ smoke were investigated. So as to macroscopically verify the filtration performance of PLA4/DTAC3, the smoke particles produced by lighted sandalwood spread quickly to occupy an enclosed box, and the box exhibited a milky white color (Figure 5f). The initial box containing the turbid smoke became transparent after 2.5 min, accompanied by the disappearance of smoke with the assistance of the circulating air, indicating the effective removal capacity of the PLA4/DTAC3 for the real PM_2.5_. In order to evaluate the filtration efficiency of the filter, the initial concentration of PM_2.5_ was quantitatively set above 900 μg·m^−3^; the content of PM_2.5_ reduced to below 10 μg·m^−3^ would be judged as one cycle. Notably, the efficient filtration ability of PLA4/DTAC3 could still be well maintained even after the consecutive 20-cycle tests, and each cycle was completed within 1.5 min (Figure 5g), indicating the excellent filtration property of PLA4/DTAC3.

### 3.6. Antibacterial Property and Heat Dissipation of the Filter

**Figure 6:**
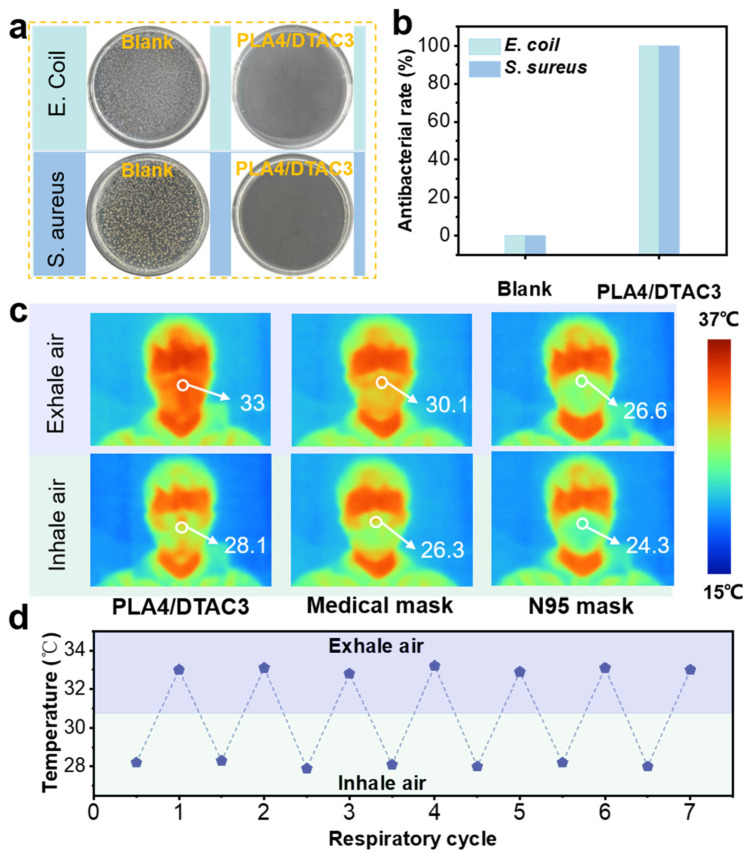
(**a**,**b**) Quantitative antibacterial properties of blank sample and PLA4/DTAC3 for resisting *S. aureus* and *E. coli*. (**c**) IR images of PLA4/DTAC3, medical mask, and N95 mask. (**d**) Surface temperatures of PLA4/DTAC3 during different respiratory cycles.

The antibacterial property is a critical parameter, as large quantities of bacteria can conceal themselves within aerosols. Even if these aerosols can be efficiently intercepted by filters, the bacteria will continue to survive on the surface of filters, thereby posing a significant threat to human respiratory health [57,58]. Therefore, the shaking method was employed to quantitatively estimate the antibacterial property of PLA4/DTAC3. Both *E. coli* and *S. aureus* exhibited robust growth without the existence of an antibacterial substance, and were uniformly distributed on a Petri dish, while the *E. coli* and *S. aureus* that were cultivated together with PLA4/DTAC3 were almost all killed (Figure 6a), achieving antibacterial ratios up to 99.9% (Figure 6b). The superior antibacterial activity of PLA4/DTAC3 could be attributed to the effect of DTAC as an antibacterial agent. The positively charged quaternary nitrogen in DTAC easily contacts the negatively charged phospholipid head groups in the bacterial cell membrane, leading to the destruction of membrane integrity and the subsequent bacterial inactivation [59]. Furthermore, as a cationic surfactant, DTAC possessed an appropriate N-alkyl chain length and favorable lipophilicity, and could induce the denaturation and degradation of structural proteins and enzymes, also contributing to its antibacterial effects [35,60,61]. Therefore, PLA4/DTAC3 can effectively exhibit antibacterial performances during wearing, thereby avoiding the potential hazards caused by the survival of bacteria on the filter surface.

While filters are commonly employed for personal protection in high-temperature environments, inadequate dissipation performance poses significant physiological challenges, which can prompt the emergence of facial erythema, heat stroke, and heat exhaustion [62]. Consequently, the thermal dissipation performance of filters is a critical parameter concerning both the comfortability and safety during wearing in high-temperature conditions [31]. To visually demonstrate the heat dissipation of the as-prepared PLA4/DTAC3, the variations in real-time temperature on its outer surface during seven respiratory cycles, including inspiratory and expiratory processes, were recorded (Figure 6c). The temperature difference between exhalation and inhalation in each breathing cycle was approximately 4.9 °C. During inhalation, the average surface temperature of PLA4/DTAC3 was 28.1 °C, while it rose to about 33 °C during exhalation. Meanwhile, the temperature differences for medical masks and N95 masks were only 3.8 °C and 2.3 °C, respectively (Figure 6d). Moreover, when wearing PLA4/DTAC3, the surface temperature during exhalation was significantly higher than that of the other commercial filters, indicating that the warm air produced during exhalation can dissipate more rapidly. The higher temperature in inhalation process compared to the other two filters was attributed to the fact that the lightweight and thin structure of the PLA4/DTAC3 made it easier for the thermal imager to capture the real-time facial temperatures.

## 4. Conclusions

In summary, this work demonstrated a novel and effective method to construct a hierarchically structured filter composed of multi-scale fibers and beads, which leads to a high filtration performance, high antibacterial performance, and rapid heat dissipation through a single-jet electrospinning process. The formulation of the cationic antibacterial surfactant in a low concentration PLA solution could regulate the ejection and splitting of the charged jets and promote the jets to stretch into nanofibers (with an average diameter of 31.5 nm), submicron-fibers (with an average diameter of 268 nm), and beads (with an average diameter of 5000 nm) simultaneously. The three-dimensional multi-modal fibers and beads can complement each other to form the average pore size of 0.34 μm, while the beads can provide a three-dimensional support, offering abundant pathways for the airflow to pass through, thereby endowing the filter with an excellent filtration efficiency of 99.979% and ultra-low pressure drop of 18.7 Pa. Notably, the long term filtration performance stability of the filter was outstanding during the various tests, including 20-cyclic and 480 min long-term tests, high-humidity tests, and actual 20-cycle PM_2.5_ smoke tests. Additionally, the cationic antibacterial surfactant enabled the filter to present remarkable antibacterial performance, while the lightweight and thin structure ensured that the filter had excellent heat dissipation properties. Therefore, the hierarchical fibrous filter, which can be efficiently prepared via a single-jet configuration in large-scale e-spun equipment, may offer a potential way to manufacture a PLA-based air filter with multidimensional protective performance and comfortable wearability.

## Data Availability

The research data were partially recorded in the Supplementary Material; the rest of the data can be obtained from the authors upon reasonable request.

## Supplementary Materials

The following supporting information can be downloaded at https://www.mdpi.com/article/10.3390/nano15181452/s1. Figure S1: SEM images of PLA made from (a) 2 wt%, (b) 4 wt%, (c) 6 wt% and (d) 8 wt%; Figure S2: FTIR spectra of PLA4, DTAC, PLA4/DTAC1, PLA4/DTAC3, PLA4/DTAC5, PLA4/DTAC7 at (a) 1200–4000 cm^−1^ and (b) 500–1200 cm^−1^; Figure S3: Filtration efficiency and pressure drop of PLA4/DTAC3 comparing with literatures Table S1: Preparation parameters of nanofibrous membranes; Table S2: Solution properties of 4 wt% PLA solutions with the varied contents of DTAC; Table S3: The basic parameters change in PLA4/DTAC3 along with the spinning time. Table S4: Filtration performance of PLA4/DTAC3 for cyclic test; Table S5: Filtration performance of PLA4/DTAC3 in long-term test; Table S6: Filtration performance of PLA4/DTAC3 under different airflow velocities. Table S7: Filtration performance of PLA4/DTAC3 placed in 60% RH; Table S8: Filtration performance of PLA4/DTAC3 placed in 90%. References [63,64,65,66,67,68] are cited in the supplementary materials.
