# Supplementary material for: Jet Splitting Enabled One-Step Fabrication of Hierarchically Structured PLA Membranes for High-Performance PM0.3 Filtration
Source: Nanomaterials (Basel). 2025 Sep 20;15(18):1452. doi: 10.3390/nano15181452 (PMC12472766; doi:10.3390/nano15181452)
Supplement: Supplementary file 1 [file nanomaterials-15-01452-s001.zip › nanomaterials-3835144-supplementary.pdf]

Supplementary Material

Jet Splitting Enabled One-step Fabrication of Hierarchically Structured PLA Membranes for High-performance $PM_{0.3}$ Filtration

Yintao Zhao [1,2,3], Ying Chen [4], Xin Ning [1,3*]

1 Industrial Research Institute of Nonwovens & Technical Textiles, College of Textiles & Clothing, Qingdao University, Qingdao 266071, Shandong, China; ZHytooo@163.com (Y.Z.); xning@qdu.edu.cn (X. N.);
2 College of Textiles & Clothing, Dezhou University, Dezhou 253026, Shandong, China. ZHytooo@163.com (Y.Z.);
3 Shandong Center for Engineered Nonwovens, Qingdao University, Qingdao 266071, Shandong, China. ZHytooo@163.com (Y.Z.); xning@qdu.edu.cn (X. N.);
4 Qingdao Institute of Food and Drug Control, Qingdao 266071, Shandong, China; cheny06301@163.com (Y. C)

* Corresponding Author

Prof. Dr. Xin Ning

College of Textiles & Clothing

Qingdao University

308 NingXia Road, Shinan District, Qingdao 266071, Shandong, China.

E-mail address: xning@qdu.edu.cn (Xin Ning);

Contents

Supplementary Material 1

1. Supporting Figures 3

Figure S1 SEM images of PLA made from (a) 2 wt%, (b) 4 wt%, (c) 6 wt% and (d) 8 wt%...3

Figure S2 FTIR spectra of PLA4, DTAC, PLA4/DTAC1, PLA4/DTAC3, PLA4/DTAC5, PLA4/DTAC7 at (a) 1200-4000 cm^{-1} and (b) 500-1200 cm^{-1}..4

Figure S3 Filtration efficiency and pressure drop of PLA4/DTAC3 comparing with literatures [1-9]..5

2. Supporting Tables 6

Table S1 Preparation parameters of nanofibrous membranes 6

Table S2 Solution properties of 4 wt% PLA solutions with the varied contents of DTAC 7

Table S3 The basic parameters change in PLA4/DTAC3 along with the spinning time. 8

Table S4 Filtration performance of PLA4/DTAC3 for cyclic test. 9

Table S5 Filtration performance of PLA4/DTAC3 in long-term test 10

Table S6 Filtration performance of PLA4/DTAC3 under different airflow velocities. 11

Table S7 Filtration performance of PLA4/DTAC3 placed in 60% RH..........................12

Table S8 Filtration performance of PLA4/DTAC3 placed in 90% RH..........................13

Reference 14

1. Supporting Figures

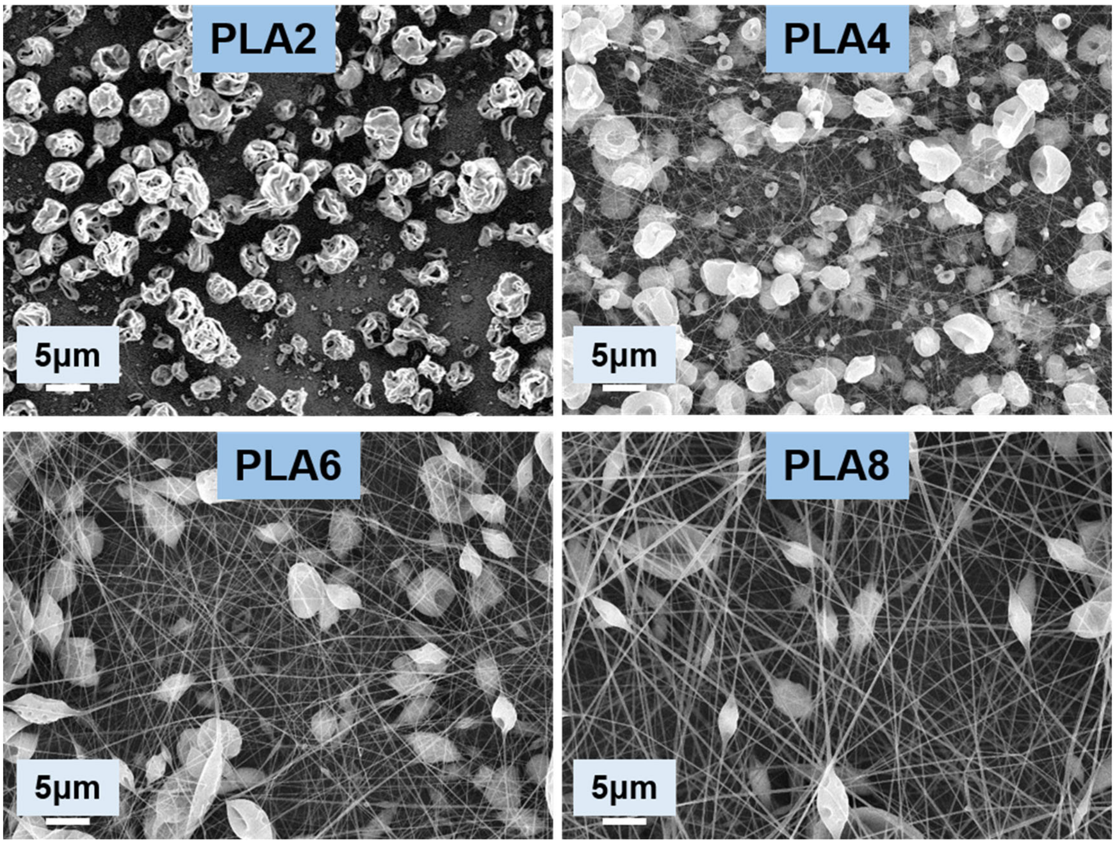

Figure S1 SEM images of PLA made from (a) 2 wt%, (b) 4 wt%, (c) 6 wt%, and (d) 8 wt%.

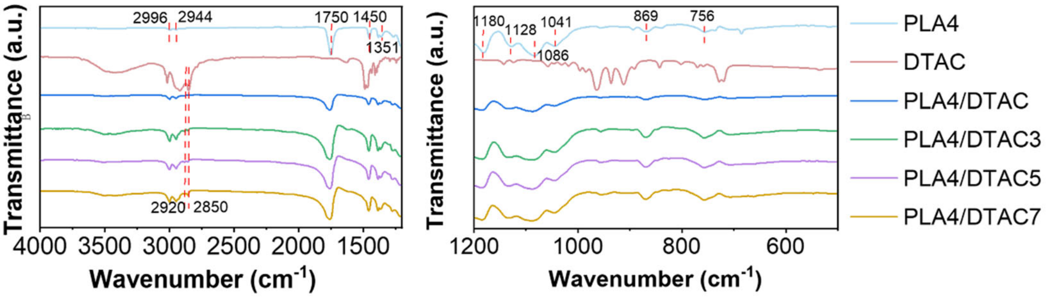

Figure S2 FTIR spectra of PLA4, DTAC, PLA4/DTAC1, PLA4/DTAC3, PLA4/DTAC5, and PLA4/DTAC7 at (a) 1200-4000 cm^{-1} and (b) 500-1200 cm^{-1}.

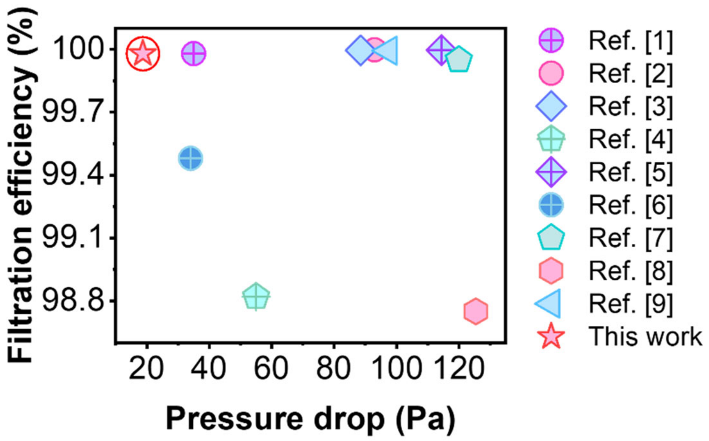

Figure S3 Filtration efficiency and pressure drop of PLA4/DTAC3 compared with the literature [1-9].

2. Supporting Tables

Table S1 Preparation parameters of nanofibrous membranes.

Samples	PLA concentration (wt%)	Mass ratio of DTAC to PLA (wt%)	Voltage (kV)	Feeding rate (mL/h)	Distance (cm)
PLA2	2	0	25	1	18
PLA4	4	0	25	1	18
PLA6	6	0	25	1	18
PLA8	8	0	25	1	18
PLA4/DTAC1	4	1	25	1	18
PLA4/DTAC3	4	3	25	1	18
PLA4/DTAC5	4	5	25	1	18
PLA4/DTAC7	4	7	25	1	18

Table S2 Solution properties of 4 wt% PLA solutions with the varied contents of DTAC.

Content of DTAC (wt%)	Viscosity (mP·s)	Conductivity (μs/cm)	Surface tension (mN/m)
0	42.92±0.03	0.56±0.02	20.58±1.08
1	42.63±0.02	46.34±0.67	20.22±1.03
3	42.12±0.05	108.58±12.73	19.86±0.82
5	41.59±0.01	152.85±2.36	19.53±1.09
7	41.23±0.06	185.25±4.18	19.06±0.84

Table S3 The basic parameter change in PLA4/DTAC3 along with the spinning time.

Spinning time (min) / Parameters	10	15	20	25
Thickness (μm)	5.15±2.13	6.89±1.86	8.96±3.4	10.9±1.44
Basic weight (g·m^{-2})	0.137±0.008	0.204±0.074	0.275±0.015	0.352±0.06
Average pore size (μm)	0.45±0.28	0.37±0.18	0.34±0.25	0.34±0.17
Filtration efficiency (%)	97.88±0.142	99.882±0.027	99.979±0.004	99.979±0.002
Pressure drop (Pa)	12.3±0.5	15.7±0.5	18.7±0.5	21.7±0.5
Quality factor (Pa^{-1})	0.312±0.005	0.43±0.015	0.444±0.009	0.391±0.005

Table S4 Filtration performance of PLA4/DTAC3 for cyclic test.

Cycle	Filtration efficiency (%)	Pressure drop (Pa)
1	99.979±0.004	18.7±0.5
2	99.963±0.003	18.7±0.5
3	99.967±0.007	18.7±0.5
4	99.965±0.007	18.7±0.5
5	99.955±0.012	19±0
6	99.955±0.013	19±0
7	99.97±0.005	19±0
8	99.98±0.0002	19±0
9	99.966±0.007	19±0
10	99.956±0.007	19.3±0.5
11	99.973±0.003	19±0
12	99.964±0.008	19±0
13	99.97±0.0002	19.3±0.5
14	99.96±0.01	19.3±0.5
15	99.961±0.009	19.3±0.5
16	99.964±0.008	19.3±0.5
17	99.961±0.009	19.3±0.5
18	99.963±0.003	19.3±0.5
19	99.971±0.005	19.3±0.5
20	99.957±0.012	19.3±0.5

Table S5 Filtration performance of PLA4/DTAC3 in long-term test.

Filtration time (min)	Filtration efficiency (%)	Pressure drop (Pa)
0	99.979±0.004	18.7±0.5
30	99.975±0.002	18.7±0.5
60	99.962±0.009	18.7±0.5
90	99.98±0.0002	18.7±0.5
120	99.977±0.001	19±0.8
150	99.972±0.004	19±0.8
180	99.979±0.0004	19±0
210	99.974±0.003	19.3±0.5
240	99.962±0.004	19.3±1.2
270	99.975±0.002	19.7±0.5
300	99.96±0.005	19.7±0.5
330	99.942±0.009	19.7±0.5
360	99.934±0.008	20±0.8
390	99.925±0.012	20±0
420	99.933±0.003	20.3±0.5
450	99.943±0.004	20.3±0.5
480	99.939±0.0005	20.3±0.5

Table S6 Filtration performance of PLA4/DTAC3 under different airflow velocities.

Airflow (L/min)	Filtration efficiency (%)	Pressure drop (Pa)
20	99.987±0.002	12.3±0.5
32	99.979±0.004	18.7±0.5
40	99.965±0.01	24.3±0.5
50	99.946±0.01	30.7±0.5
60	99.926±0.016	36±0.8
70	99.885±0.023	42±0
80	99.845±0.004	48.3±0.5
90	99.747±0.027	54.3±0.5
100	99.793±0.037	60.3±0.5
110	99.746±0.032	65.7±0.5
120	99.747±0.059	71.7±0.5

Table S7 Filtration performance of PLA4/DTAC3 placed in 60% RH.

Placed time (h)	Filtration efficiency (%)	Pressure drop (Pa)
0	99.979±0.004	18.7±0.5
3	99.979±0.003	18.7±0.5
6	99.965±0.006	18.7±0.5
9	99.898±0.007	18.3±0.5
12	99.936±0.005	18.7±0.5
15	99.934±0.012	18.7±0.5
18	99.926±0.011	18.7±0.5
21	99.956±0.008	18.3±0.5
24	99.953±0.008	18.7±0.5

Table S8 Filtration performance of PLA4/DTAC3 placed in 90% RH.

Placed time (h)	Filtration efficiency (%)	Pressure drop (Pa)
0	99.979±0.004	18.7±0.5
3	99.896±0.017	18.7±0.5
6	99.894±0.012	18.7±0.5
9	99.787±0.033	18.7±0.5
12	99.708±0.029	18.3±0.5
15	99.536±0.03	18.3±0.5
18	99.544±0.036	18.7±0.5
21	99.59±0.035	18.7±0.5
24	99.6±0.034	19±0.8

Reference

1 Yang Y, Yang Y, Huang J, Li S, Meng Z, Cai W, Lai Y. Electrospun Nanocomposite Fibrous Membranes for Sustainable Face Mask Based on Triboelectric Nanogenerator with High Air Filtration Efficiency. Adv. Fiber Materials. 2023, 5, 1505.

2 Liu H, Zhang S, Liu L, Yu J, Ding B. High-performance PM0. 3 air filters using self-polarized electret nanofiber/nets. Adv. Func. Materials. 2020 Mar;30(13):1909554.

3 Zhang S, Liu H, Tang N, Zhou S, Yu J, Ding B. Spider-web-inspired PM0. 3 filters based on self-sustained electrostatic nanostructured networks. Adv. Mater. 2020 Jul;32(29):2002361.

4 Dong T, Hua Y, Zhu X, Huang X, Chi S, Liu Y, Lou CW, Lin JH. Highly efficient and sustainable PM filtration using piezo nanofibrous membrane with gradient shrinking porous network. *Sep. Purif. Technol*. 2022 May 15;289:120753.

5 Xu R, Feng J, Zhang L, Li S. Low viscosity of spinning liquid to prepare organic-inorganic hybrid ultrafine nanofiber membrane for high-efficiency filtration application. *Sep. Purif. Technol*. 2022 Dec 15;303:122224.

6 Yang M, Li X, Yao N, Yu J, Yin X, Zhang S, Ding B. Two-dimensional piezoelectric nanofibrous webs by self-polarized assembly for high-performance PM0. 3 filtration. ACS nano. 2024 Jun 21;18(26):16895-904.

7. Yang Y, Li X, Zhou Z, Qiu Q, Chen W, Huang J, Cai W, Qin X, Lai Y. Ultrathin, ultralight dual-scale fibrous networks with high-infrared transmittance for high-performance, comfortable and sustainable PM0. 3 filter. Nat. Commun. 2024 Feb 21;15(1):1586.

8. Liu Y, Jia C, Zhang H, Wang H, Li P, Jia L, Wang F, Zhu P, Wang H, Yu L, Wang F. Free-standing ultrafine nanofiber papers with high PM0. 3 mechanical filtration efficiency by scalable blow and electro-blow spinning. *ACS Appl. Mater. Interfaces*. 2021 Jul 19;13(29):34773-81.

9 Gao H, Li J, Fu H, Zhang K, Duan S, Zhao H, Zheng Z, Zhou M, Xia Z, Liu Y. Deep trapped bipolar heterocharges enable electret nanofibrous membranes for high-efficiency PM0.3 filtration. *Sep. Purif. Technol*. 2025, 354, 128931.
